# Programming Native CRISPR Arrays for the Generation of Targeted Immunity

**DOI:** 10.1128/mBio.00202-16

**Published:** 2016-05-03

**Authors:** Alexander P. Hynes, Simon J. Labrie, Sylvain Moineau

**Affiliations:** Département de Biochimie, de Microbiologie et de Bio-informatique, Université Laval, Québec, Canada

## Abstract

The adaptive immune system of prokaryotes, called CRISPR-Cas (clustered regularly interspaced short palindromic repeats and CRISPR-associated genes), results in specific cleavage of invading nucleic acid sequences recognized by the cell’s “memory” of past encounters. Here, we exploited the properties of native CRISPR-Cas systems to program the natural “memorization” process, efficiently generating immunity not only to a bacteriophage or plasmid but to any specifically chosen DNA sequence.

## IMPORTANCE

CRISPR-Cas systems have entered the public consciousness as genome editing tools due to their readily programmable nature. In industrial settings, natural CRISPR-Cas immunity is already exploited to generate strains resistant to potentially disruptive viruses. However, the natural process by which bacteria acquire new target specificities (adaptation) is difficult to study and manipulate. The target against which immunity is conferred is selected stochastically. By biasing the immunization process, we offer a means to generate customized immunity, as well as provide a new tool to study adaptation.

## OBSERVATION

The microbial adaptive immune system, called CRISPR-Cas (clustered regularly interspaced short palindromic repeats and CRISPR-associated genes), results in specific cleavage of invading nucleic acid sequences recognized by the cell’s “memory” of past encounters (1). The ability of the system to adapt and offer protection against previously unencountered invaders is what enables it to be readily “programmed” with customized RNA guides. This, in turn, has led to the use of CRISPR-Cas as an exceptional tool for directed genome editing (2). The natural process of adaptation in CRISPR-Cas systems, however, involves the incorporation of short (~30-nucleotide [nt]) spacers, typically derived from foreign genetic elements, into a repeat-spacer array (CRISPR) that forms the memory of the system (1). These spacers derived from foreign sequences are known as protospacers. The only known sequence constraint on what can serve as a protospacer is adjacency to a PAM (protospacer-adjacent motif)—a short (~3- to 7-nt) recognition motif specific to any given type I or type II CRISPR-Cas system. This PAM is required for both acquisition of a new spacer and subsequent cleavage of a targeted protospacer (3). Transcription of the “memory” array generates CRISPR RNA (crRNA) guides (4) that, in complex with a variety of Cas proteins, act as surveillance complexes that recognize and cleave matching invading sequences (5).

The natural process of adaptation has proven difficult to study. Very few organisms to date have demonstrated readily detectable spacer acquisition (3, 6–9). Even when spacer acquisition is evident, such as in the Gram-positive bacterium *Streptococcus thermophilus*, it is seemingly stochastic. When *S. thermophilus* cells are challenged with virulent phages, approximately one in 10^6^ cells survive by acquisition of a new phage-derived spacer within the CRISPR array (10); however, any of the phage protospacers (e.g., 716 in the genome of the *S. thermophilus* phage 2972 [11]) could form the basis of that immunity. Engineering this immunity is complicated, as the CRISPR loci are generally difficult to manipulate. The nature of the repeat structures makes synthesis of oligonucleotides difficult and recombination unpredictable. This is generally avoided by the creation of custom CRISPR arrays borne on plasmid vectors (12–14). This option has to be tailored to each CRISPR-Cas system, is ill-suited for strains with few suitable vectors, and carries with it the pitfalls generally associated with nonchromosomal, higher-copy-number systems. Once generated, however, the resulting constructs have proven useful for manipulating genetic material *in vivo*, facilitating previously laborious tasks like the editing of virulent phage genomes (13).

In order to better manipulate the native CRISPR arrays, we have to understand any biases in spacer acquisition. Recently, three such biases (or lack thereof) were uncovered: a preference for acquisition from defective phages (15), as well as no preference for the targeting of nonself DNA elements in the absence of selection (16), with the exception of enrichment from stalled replication forks and associated DNA damage (17). Together, these findings suggest that plasmids are highly preferred targets for spacer acquisition. In fact, for the purposes of the CRISPR-Cas system, plasmids are analogous to defective phages in that there is no race to acquire a spacer before suffering irreparable cell damage. As plasmids can be present in high copy numbers, the number of protospacer targets increases. Furthermore, the acquisition of a spacer from a plasmid leads to the loss of that plasmid, which is generally associated with a direct fitness benefit to the bacterium.

**FIG 1:**
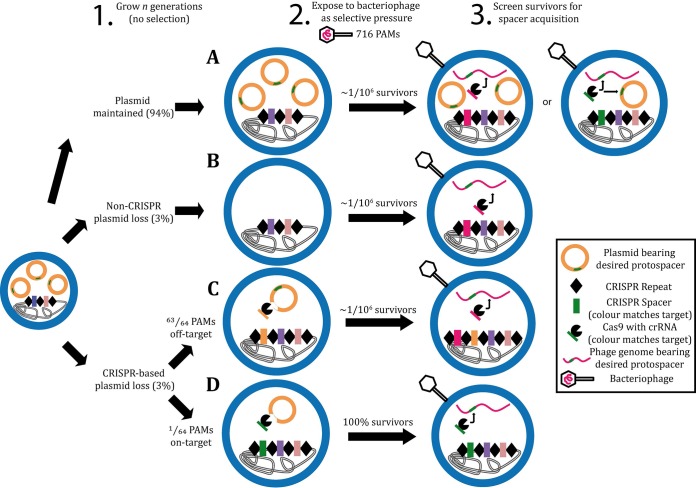
Programming a native CRISPR array. Bacterial growth in the absence of selection for the plasmid bearing the desired protospacer (step 1) results in one of four scenarios. In scenario A, the plasmid is maintained and exposure to a virulent phage (step 2) results in typical CRISPR immunization, with one in 10^6^ survivors (step 3). There could be a moderate bias toward acquisition of the desired spacer, as it is more abundant (high-copy-number plasmid) than any other immunity-conferring protospacer. In scenario B or C, the plasmid is either lost through acquisition of a plasmid-targeting spacer other than the desired one (scenario C) or by other means (scenario B). When exposed to phages, these cells are only capable of typical naive CRISPR immunization, with one in 10^6^ survivors having randomly acquired 1 of the 716 possible phage-derived spacers. It is possible that the fitness benefit of curing the plasmid has enriched the population for cells more prone to CRISPR acquisition, offering an increase in the immunization rate. In scenario D, the plasmid is lost due to acquisition of the desired plasmid-borne, phage-derived protospacer. While this event should be rare (one of the 64 protospacers on the plasmid is the desired one), all cells that have acquired this spacer will survive exposure to the phages. These should be a considerable proportion of the colonies surviving phage exposure.

Here, we exploit the acquisition of spacers from plasmids as a tool to bias (and select for) the natural acquisition of specific spacers—in other words, readily programming the native CRISPR arrays. Designing a custom protospacer for inclusion on a plasmid, we allow time for plasmid loss by spacer acquisition within the CRISPR locus (Fig. 1, step 1) and then, by adding selection via a phage bearing the desired protospacer (Fig. 1, step 2), we select for survivors with CRISPR-conferred immunity. The resulting phage-resistant colonies should have preferentially picked up the desired spacer cloned on the plasmid. Where the resulting bias is insufficient, a number of parameters may be easily manipulated: (i) increasing the number of generations for plasmid loss, (ii) increasing the benefit to the cell of losing the plasmid—ideally by using a higher-copy-number vector, which may also further bias spacer selection by the abundance of the desired target, and (iii) applying a screen for plasmid loss at step 2 (or 3) and thus enriching the population of desired spacers.

The low-copy-number plasmid pNT1 had previously been shown to be cured by the two active type II-A CRISPR-Cas systems (CR1 and CR3) of *S. thermophilus* DGCC7710 (5). In that study, 6% of cells screened after 60 generations had lost the plasmid, 55% of those due to the acquisition of a plasmid-targeting spacer within a CRISPR array. While this would be suitable, we hypothesized that the additional replicative burden of the high-copy-number (>50) (18) plasmid pNZ123 would result in faster plasmid loss. When we introduced the high-copy-number plasmid pNZ123 into *S. thermophilus* DGCC7710, we observed plasmid loss in 6.5% (41/623) of screened colonies after only seven generations. We checked 18 colonies for spacer acquisition, and 11 of them (61%) had acquired one of eight different plasmid-specific spacers at either the CR1 locus (10 colonies) or the CR3 locus (1 colony). Clearly, spacers can be readily and rapidly acquired from this high-copy-number plasmid.

To program the native CRISPR array to acquire a specific desired spacer from a plasmid and as proof of concept, we sought to introduce a spacer that would provide resistance against several phages.

In *S. thermophilus* DGCC7710, the CR1 locus is responsible for >90% of spacer acquisition events (19) and requires recognition of an NNAGAAW PAM (3). The other active locus, CR3, depends upon recognition of a shorter PAM, NGGNG (20). We scanned the 13 publicly available *S. thermophilus* phage genomes for identical 30-bp, PAM-adjacent sequences (i.e., protospacers). We found a number of protospacers that were conserved in over half of these genomes and even across two distinct phage groups (Fig. S1 in the supplemental material). Decreasing the length of the required PAM-adjacent sequence match to as few as 15 bp increased the number of protospacers detected in at least seven genomes but yielded only a single protospacer matching more than seven (see Fig. S1). With further characterization of the “seed” sequences (minimal regions of the protospacer absolutely required for immunity [13, 21]), it should be possible to design protospacers with even broader cross-immunity. We selected both a CR1 and a CR3 protospacer (see Fig. S1 and Table S1), targeting the greatest number of virulent phages in a highly conserved gene less likely to be tolerant of mutations. Both protospacers and their respective PAMs were cloned into pNZ123 to generate the programming plasmids pNZCR1 and pNZCR3.

*S. thermophilus* DGCC7710 cells containing the control plasmid (pNZ123) or a programming plasmid (pNZCR1 or pNZCR3) were grown in the absence of selection for the plasmid and then exposed to the virulent phage 2972. Strikingly, more than 100 times as many cells carrying a programming plasmid survived phage infection as did cells carrying the control plasmid (123 times as many for pNZCR3 and 419 times as many for pNZCR1) (Table 1). This indicated that the phage-immune cells obtained were influenced by the presence of the chosen phage-derived, plasmid-borne protospacer.

**TABLE 1:** Counts and characterization of surviving colonies in CRISPR-programming assays

Strain (plasmid)	Dilution factor^a^	Mean no. of survivors (CFU/plate) ± SD (*n* = 3)	No. of survivors (CFU/ml)	Fraction surviving	Acquisition profile (no. of colonies with acquisition at indicated locus/no. of colonies tested)	No. of on-target acquisitions/total no. of colonies tested
DGCC7710 (pNZ)	NA	56.0 ± 11.4	187	1.85 in 10^6^ CFU	23/24 CR1, 1/24 CR3	0/24
DGCC7710 (pNZCR3)	1/9	764 ± 18.9	22,940	2.27 in 10^4^ CFU	4/24 CR1, 20/24 CR3	20/24 (100% of CR3)
DGCC7710 (pNZCR1)	1/27	868 ± 14.3	78,168	7.73 in 10^4^ CFU	24/24 CR1, 0/24 CR3	24/24 (100% of CR1)

a Dilution factor (in fresh media) used in plate from which colony counts were obtained (vol/vol).

To confirm this, the CRISPR loci of phage-resistant colonies were screened by PCR in order to detect integration of the target spacers. Where such a spacer was not detected, the CR1 and CR3 loci were amplified to detect expansion of the arrays by a repeat-spacer unit (66 bp) and then sequenced. For phage-resistant cells carrying the control plasmid pNZ123, spacer acquisition from the phage genome occurred but appeared to be stochastic. The spacer acquisition patterns matched the expected CR1-to-CR3 natural bias for *S. thermophilus* DGCC7710 (Table 1). Unsurprisingly, none of the phage-resistant colonies tested (0/24) had acquired the spacers present in the programming plasmids pNZCR1 and pNZCR3.

In the presence of the programming plasmid pNZCR1, 100% (24/24) of the phage-resistant colonies tested had acquired the desired phage-derived spacer present on the plasmid. Despite the documented lower adaptation activity of CR3 in *S. thermophilus* DGCC7710 (19), in the presence of pNZCR3, 20/24 colonies tested had acquired the desired spacer (83.3%) (Table 1). This represents over a thousandfold (1,024) increase in CR3-resistant colonies over the numbers obtained in unprogrammed assays. By both replica plating of colonies in the absence/presence of chloramphenicol and a PCR screen for the vector, we confirmed that the pNZCR1 and pNZCR3 plasmids were absent in all phage-resistant survivors bearing the target spacers.

Plasmid-programed spacer acquisition is a quick, inexpensive, and labor-light method to select for the integration of specific spacers into native CRISPR arrays. Furthermore, the resulting product is clean and markerless, with no trace of the plasmid used to bias the selection process—meaning that it is readily repeatable with a single vector and indistinguishable from natural acquisition.

Constraining the application of this strategy are the following three requirements: a host capable of spacer acquisition and subsequent interference, a moderately stable/persistent vector, and the dependence of selective pressure on the replication of a genetic element bearing, or edited to bear, the desired protospacer. Because we can easily edit conditionally lethal plasmids or phage genomes (13) to incorporate these targets, when these three requirements are met, we can readily obtain the integration of any desired sequence of the appropriate size into a CRISPR array.

This approach lends itself to many applications. A key example from industrial settings such as dairy fermentations is the standard practice of generating spontaneously phage-resistant bacterial cultures (22). Accordingly, the generation of spontaneous CRISPR-Cas-based immunity is already widely used for *S. thermophilus* (23), but because spacers are acquired stochastically, any attempt to obtain a specific spacer must entail excessive screening. Instead of this inefficient process, we have shown here the rapid generation of customized bacterial strains immune to multiple phages, even prior to exposure to (or even discovery of) said phages, by targeting conserved protospacers. Similarly, it would be possible to create beneficial strains refractory to a plasmid/antibiotic resistance gene, serving to reduce horizontal gene transfer of undesirable genes or, alternatively, specifically select for functional sequence variants.

Applications in basic CRISPR-Cas research include studying spacer acquisition events at loci that rarely acquire them (e.g., CR3 in in *S. thermophilus* DGCC7710) or are thought to be defective. This technique also allows the determination of optimal features of both PAMs and protospacers by varying the two components independently of one another, screening for functional PAMs or other CRISPR-related motifs, and assaying their activities for acquisition and interference. Lastly, it could be used to insert specific sequences at the CRISPR loci, such as promoters, terminators, DNA binding sites, or additional CRISPR repeats capable of modulating or serving as reporters for CRISPR acquisition.

This “spacer on demand” strategy provides a simple and novel means to introduce any chosen spacer into a native CRISPR array. This offers new tools to characterize CRISPR-Cas systems and to exploit them to generate a resistance phenotype with unprecedented flexibility.

## SUPPLEMENTAL MATERIAL

Figure S1  Shared protospacer detection for all 13 complete *S. thermophilus* phage genomes currently available in public databases. The matrix (top) highlights the similarity of any phage to all others, as calculated by attributing increasing relatedness (darker color) based on the number of protein homologues they share (see Text S1). By this method, *S. thermophilus* phages separate into at least three clearly distinct groups, identified by blue, white (i.e., no shading), or red shading. The table (bottom) displays all protospacers shared by the greatest number of phage genomes (≥7), and their presence (blue) or absence (white) from each phage genome. The boxed rows indicate the two protospacers chosen for this study. S, shortened spacer; querying our database with shorter and shorter spacer lengths, to a minimum of 15, only yielded one candidate with homology to more than 7 database phages. Download Figure S1, DOCX file, 0.3 MB

Table S1  Strains, plasmids, and oligonucleotides used in this study. Table S1, DOCX file, 0.1 MB

Text S1  Methods used in this study. Download Text S1, DOCX file, 0.3 MB

## References

[1] Barrangou R, Fremaux C, Deveau H, Richards M, Boyaval P, Moineau S, Romero DA, Horvath P 2007 CRISPR provides acquired resistance against viruses in prokaryotes. Science 315:1709–1712. doi:10.1126/science.1138140. 17379808

[2] Jinek M, Chylinski K, Fonfara I, Hauer M, Doudna JA, Charpentier E 2012 A programmable dual-RNA-guided DNA endonuclease in adaptive bacterial immunity. Science 337:816–821. doi:10.1126/science.1225829. 22745249 PMC6286148

[3] Deveau H, Barrangou R, Garneau JE, Labonté J, Fremaux C, Boyaval P, Romero DA, Horvath P, Moineau S 2008 Phage response to CRISPR-encoded resistance in *Streptococcus thermophilus*. J Bacteriol 190:1390–1400. doi:10.1128/JB.01412-07. 18065545 PMC2238228

[4] Deltcheva E, Chylinski K, Sharma CM, Gonzales K, Chao Y, Pirzada ZA, Eckert MR, Vogel J, Charpentier E 2011 CRISPR RNA maturation by trans-encoded small RNA and host factor RNase III. Nature 471:602–607. doi:10.1038/nature09886. 21455174 PMC3070239

[5] Garneau JE, Dupuis MÈ, Villion M, Romero DA, Barrangou R, Boyaval P, Fremaux C, Horvath P, Magadán AH, Moineau S 2010 The CRISPR/Cas bacterial immune system cleaves bacteriophage and plasmid DNA. Nature 468:67–71. doi:10.1038/nature09523. 21048762

[6] Li M, Wang R, Zhao D, Xiang H 2014 Adaptation of the *Haloarcula hispanica* CRISPR-Cas system to a purified virus strictly requires a priming process. Nucleic Acids Res 42:2483–2492. doi:10.1093/nar/gkt1154. 24265226 PMC3936756

[7] Cady KC, Bondy-Denomy J, Heussler GE, Davidson AR, O’Toole GA 2012 The CRISPR/Cas adaptive immune system of *Pseudomonas aeruginosa* mediates resistance to naturally occurring and engineered phages. J Bacteriol 194:5728–5738. doi:10.1128/JB.01184-12. 22885297 PMC3486085

[8] Erdmann S, Garrett RA 2012 Selective and hyperactive uptake of foreign DNA by adaptive immune systems of an archaeon via two distinct mechanisms. Mol Microbiol 85:1044–1056. doi:10.1111/j.1365-2958.2012.08171.x. 22834906 PMC3468723

[9] Richter C, Dy RL, McKenzie RE, Watson BNJ, Taylor C, Chang JT, McNeil MB, Staals RHJ, Fineran PC 2014 Priming in the type I-F CRISPR-Cas system triggers strand-independent spacer acquisition, bi-directionally from the primed protospacer. Nucleic Acids Res 42:8516–8526. doi:10.1093/nar/gku527. 24990370 PMC4117759

[10] Levin BR, Moineau S, Bushman M, Barrangou R 2013 The population and evolutionary dynamics of phage and bacteria with CRISPR-mediated immunity. PLoS Genet 9:e1003312. doi:10.1371/journal.pgen.1003312. 23516369 PMC3597502

[11] Lévesque C, Duplessis M, Labonté J, Labrie S, Fremaux C, Tremblay D, Moineau S 2005 Genomic organization and molecular analysis of virulent bacteriophage 2972 infecting an exopolysaccharide-producing *Streptococcus thermophilus* strain. Appl Environ Microbiol 71:4057–4068. doi:10.1128/AEM.71.7.4057-4068.2005. 16000821 PMC1169050

[12] Díez-Villaseñor C, Guzmán NM, Almendros C, García-Martínez J, Mojica FJ 2013 CRISPR-spacer integration reporter plasmids reveal distinct genuine acquisition specificities among CRISPR-Cas I-E variants of *Escherichia coli*. RNA Biol 10:792–802. doi:10.4161/rna.24023. 23445770 PMC3737337

[13] Martel B, Moineau S 2014 CRISPR-Cas: an efficient tool for genome engineering of virulent bacteriophages. Nucleic Acids Res 42:9504–9513. doi:10.1093/nar/gku628. 25063295 PMC4132740

[14] Jiang W, Bikard D, Cox D, Zhang F, Marraffini LA 2013 RNA-guided editing of bacterial genomes using CRISPR-Cas systems. Nat Biotechnol 31:233–239. doi:10.1038/nbt.2508. 23360965 PMC3748948

[15] Hynes AP, Villion M, Moineau S 2014 Adaptation in bacterial CRISPR-Cas immunity can be driven by defective phages. Nat Commun 5:4399. doi:10.1038/ncomms5399. 25056268

[16] Wei Y, Terns RM, Terns MP 2015 Cas9 function and host genome sampling in type II-A CRISPR–Cas adaptation. Genes Dev 29:356–361. doi:10.1101/gad.257550.114. 25691466 PMC4335292

[17] Levy A, Goren MG, Yosef I, Auster O, Manor M, Amitai G, Edgar R, Qimron U, Sorek R 2015 CRISPR adaptation biases explain preference for acquisition of foreign DNA. Nature 520:505–510. doi:10.1038/nature14302. 25874675 PMC4561520

[18] De Vos W 1987 Gene cloning and expression in lactic streptococci. FEMS Microbiol Lett 46:281–295. doi:10.1016/0378-1097(87)90113-3.

[19] Magadán AH, Dupuis MÈ, Villion M, Moineau S 2012 Cleavage of phage DNA by the *Streptococcus thermophilus* CRISPR3-cas system. PLoS One 7:e40913. doi:10.1371/journal.pone.0040913. 22911717 PMC3401199

[20] Horvath P, Romero DA, Coûté-Monvoisin A-C, Richards M, Deveau H, Moineau S, Boyaval P, Fremaux C, Barrangou R 2008 Diversity, activity, and evolution of CRISPR loci in *Streptococcus thermophilus*. J Bacteriol 190:1401–1412. doi:10.1128/JB.01415-07. 18065539 PMC2238196

[21] Hsu PD, Scott DA, Weinstein JA, Ran FA, Konermann S, Agarwala V, Li Y, Fine EJ, Wu X, Shalem O, Cradick TJ, Marraffini LA, Bao G, Zhang F 2013 DNA targeting specificity of RNA-guided Cas9 nucleases. Nat Biotechnol 31:827–832. doi:10.1038/nbt.2647. 23873081 PMC3969858

[22] Coffey A, Ross RP 2002 Bacteriophage-resistance systems in dairy starter strains: molecular analysis to application. Antonie van Leeuwenhoek 82:303–321. doi:10.1023/A:1020639717181. 12369198

[23] Horvath P, Gasiunas G, Siksnys V, Barrangou R 2013 Applications of the versatile CRISPR-Cas systems, p 267–286. In Barrangou R, van der Oost J (ed), CRISPR-Cas systems: RNA-mediated adaptive immunity in Bacteria and Archaea. Springer, Berlin, Germany.

