# Supplementary material for: Programming Native CRISPR Arrays for the Generation of Targeted Immunity
Source: mBio. 2016 May 3;7(3):e00202-16. doi: 10.1128/mBio.00202-16 (PMC4959665; doi:10.1128/mBio.00202-16)
Supplement: Figure S1 — Shared protospacer detection for all 13 complete S. thermophilus phage genomes currently available in public databases. The matrix (top) highlights the similarity of any phage to all others, as calculated by attributing increasing relatedness (darker color) based on the number of protein homologues they share (see Text S1). By this method, S. thermophilus phages separate into at least three clearly distinct groups, identified by blue, white (i.e., no shading), or red shading. The table (bottom) displays all protospacers shared by the greatest number of phage genomes (≥7), and their presence (blue) or absence (white) from each phage genome. The boxed rows indicate the two protospacers chosen for this study. S, shortened spacer; querying our database with shorter and shorter spacer lengths, to a minimum of 15, only yielded one candidate with homology to more than 7 database phages. Download [file mbo002162806sf1.docx]

**Figure S1:** Shared protospacer detection for all 13 complete *S. thermophilus* phage genomes currently available in public databases. The matrix (above) highlights the similarity of any phage to all others, as calculated by attributing increasing relatedness (darker colour) based on the number of protein ‘homologues’ they share (see Methods). By this method, *S. thermophilus* phages separate into at least three clearly distinct groups, identified by blue, white (none) or red shading. The table (below) displays all protospacers shared by the greatest number of phage genomes (≥7), and their presence (blue) or absence (white) from each phage genome. The bolded row outline indicates the two protospacers chosen for this study. S = Shortened spacer; querying our database with shorter and shorter spacer lengths, to a minimum of 15, only yielded one candidate with homology to more than 7 database phages.

**Table S1:** Strains, plasmids and oligos used in this study

| *Strains* | Species | Description | Source |
| --- | --- | --- | --- |
| NEB5α | *Escherichia coli* | Competent cells, cloning | NEB |
| DGCC7710 | *Streptococcus thermophilus* | Active & adaptive CRISPRs | (1) |
| SMQ-1333 | *E. coli* | NEB5α (pNZCR1), Cm^R^ | This study |
| SMQ-1334 | *S. thermophilus* | DGCC7710 (pNZCR1), Cm^R^ | This study |
| SMQ-1335 | *S. thermophilus* | DGCC7710 w/target CR1 spacer | This study |
| SMQ-1336 | *E. coli* | NEB5α (pNZCR3), Cm^R^ | This study |
| SMQ-1337 | *S. thermophilus* | DGCC7710 (pNZCR3), Cm^R^ | This study |
| SMQ-1338 | *S. thermophilus* | DGCC7710 w/target CR3 spacer | This study |
| SMQ-1339 | *S. thermophilus* | DGCC7710 (pNZ123), Cm^R^ | This study |
| *Plasmids* | Description | Function | Source |
| pNZ123 | Native vector, encodes chloramphenicol resistance | Negative control | (18) |
| pNZCR1 | pNZ123 with both CR1 oligos ligated in XhoI/EcoRI cut sites | CR1 programming | This study |
| pNZCR3 | pNZ123 with both CR3 oligos ligated in XhoI/EcoRI cut sites | CR3 programming | This study |
| *Oligos* | Sequence 5’-3’ | Function | Source |
| pNZins_F | AATGTCACTAACCTGCCC | pNZ123 insert screening | This study |
| pNZins_R | CATTGAACATGCTGAAGA | pNZ123 insert screening | This study |
| Forf37_CR1 | **TCGA**AGAAGCACCTCTTGCGTTGATAAAAGTATTGCAGAAA | pNZCR1 generation | This study |
| Rorf37_CR1 | **AATT**TTTCTGCAATACTTTTATCAACGCAAGAGGTGCTTCT | pNZCR1 generation, screening | This study |
| Forf37_CR3 | **TCGA**CCAATGACTGAAAACGACATTCGGAGGGTGTGGCG | pNZCR3 generation | This study |
| Rorf37_CR3 | **AATT**CGCCACACCCTCCGAATGTCGTTTTCAGTCATTGG | pNZCR3 generation, screening | This study |
| CR3-fwd | CTGAGATTAATAGTGCGATTACG | CR3 locus screening | (20) |
| CR3-rev | GCTGGATATTCGTATAACATGTC | CR3 locus screening | (20) |
| Yc70 | TGCTGAGACAACCTAGTCTCTC | CR1 locus screening | (20) |
| RDS7rev | GGATCCGGATCCGTTGAGGCCTTGTTC | CR1 locus screening | (5) |

**Bolded** text indicates overlaps to facilitate ligation into chosen site. Underlined text highlights PAM.

Cm^R^ is chloramphenicol resistance.

**References (Supplemental):**

24 **Cock PJA, Antao T, Chang JT, Chapman BA, Cox CJ, Dalke A, Friedberg I, Hamelryck T, Kauff F, Wilczynski B, de Hoon MJL**. 2009. Biopython: freely available Python tools for computational molecular biology and bioinformatics. Bioinformatics **25**:1422–1423.

25 **Kristensen DM, Kannan L, Coleman MK, Wolf YI, Sorokin A, Koonin EV, Mushegian A**. 2010. A low-polynomial algorithm for assembling clusters of orthologous groups from intergenomic symmetric best matches. Bioinformatics **26**:1481–1487.

26 R: A language and environment for statistical computing (R Foundation for Statistical Computing, Vienna, Austria, 2011).

27 gplots: Various R programming tools for plotting data, R package version 2.14.0.

28 **Holo H, Nes IF.** 1989. High-frequency transformation, by electroporation, of *Lactococcus lactis* subsp. *cremoris* grown with glycine in osmotically stabilized media. Appl Environ Microb **55**:3119-3123.
