# Supplementary material for: Programming Native CRISPR Arrays for the Generation of Targeted Immunity
Source: mBio. 2016 May 3;7(3):e00202-16. doi: 10.1128/mBio.00202-16 (PMC4959665; doi:10.1128/mBio.00202-16)
Supplement: Table S1 — Strains, plasmids, and oligonucleotides used in this study. [file mbo002162806st1.docx]

**Table S1:** Strains, plasmids and oligos used in this study

| *Strains* | Species | Description | Source |
| --- | --- | --- | --- |
| NEB5α | *Escherichia coli* | Competent cells, cloning | NEB |
| DGCC7710 | *Streptococcus thermophilus* | Active & adaptive CRISPRs | (1) |
| SMQ-1333 | *E. coli* | NEB5α (pNZCR1), Cm^R^ | This study |
| SMQ-1334 | *S. thermophilus* | DGCC7710 (pNZCR1), Cm^R^ | This study |
| SMQ-1335 | *S. thermophilus* | DGCC7710 w/target CR1 spacer | This study |
| SMQ-1336 | *E. coli* | NEB5α (pNZCR3), Cm^R^ | This study |
| SMQ-1337 | *S. thermophilus* | DGCC7710 (pNZCR3), Cm^R^ | This study |
| SMQ-1338 | *S. thermophilus* | DGCC7710 w/target CR3 spacer | This study |
| SMQ-1339 | *S. thermophilus* | DGCC7710 (pNZ123), Cm^R^ | This study |
| *Plasmids* | Description | Function | Source |
| pNZ123 | Native vector, encodes chloramphenicol resistance | Negative control | (18) |
| pNZCR1 | pNZ123 with both CR1 oligos ligated in XhoI/EcoRI cut sites | CR1 programming | This study |
| pNZCR3 | pNZ123 with both CR3 oligos ligated in XhoI/EcoRI cut sites | CR3 programming | This study |
| *Oligos* | Sequence 5’-3’ | Function | Source |
| pNZins_F | AATGTCACTAACCTGCCC | pNZ123 insert screening | This study |
| pNZins_R | CATTGAACATGCTGAAGA | pNZ123 insert screening | This study |
| Forf37_CR1 | **TCGA**AGAAGCACCTCTTGCGTTGATAAAAGTATTGCAGAAA | pNZCR1 generation | This study |
| Rorf37_CR1 | **AATT**TTTCTGCAATACTTTTATCAACGCAAGAGGTGCTTCT | pNZCR1 generation, screening | This study |
| Forf37_CR3 | **TCGA**CCAATGACTGAAAACGACATTCGGAGGGTGTGGCG | pNZCR3 generation | This study |
| Rorf37_CR3 | **AATT**CGCCACACCCTCCGAATGTCGTTTTCAGTCATTGG | pNZCR3 generation, screening | This study |
| CR3-fwd | CTGAGATTAATAGTGCGATTACG | CR3 locus screening | (20) |
| CR3-rev | GCTGGATATTCGTATAACATGTC | CR3 locus screening | (20) |
| Yc70 | TGCTGAGACAACCTAGTCTCTC | CR1 locus screening | (20) |
| RDS7rev | GGATCCGGATCCGTTGAGGCCTTGTTC | CR1 locus screening | (5) |

**Bolded** text indicates overlaps to facilitate ligation into chosen site. Underlined text highlights PAM. Cm^R^ is chloramphenicol resistance.
