# Supplementary material for: Programming Native CRISPR Arrays for the Generation of Targeted Immunity
Source: mBio. 2016 May 3;7(3):e00202-16. doi: 10.1128/mBio.00202-16 (PMC4959665; doi:10.1128/mBio.00202-16)
Supplement: Text S1 — Methods used in this study. Download [file mbo002162806s1.docx]

**Methods [Supplemental]**

*Plasmid loss assays*

*S. thermophilus* DGCC7710 with the empty vector (pNZ123) was grown overnight in M17 broth (Oxoid, Ontario, Canada) with 0.5 % w/v lactose (LM17) and 10 μg/ml chloramphenicol (CM 10) at 37 °C. Then, 100 μl of the overnight culture was used to inoculate four biological replicates of LM17 broth culture (selection-free). Once the cultures reached an OD_600_ of 0.6 (~7 generations), the cultures were serially diluted in fresh media, plated on LM17 and incubated overnight at 37°C. Single colonies were then individually picked and transferred into 96-well plates with LM17 media as well as plates with LM17 Cm 10, and then left to grow overnight at 37 °C. Any well which had supported grown in LM17 but not in LM17 Cm 10 was considered a plasmid loss event, and a subset of those were screened for expansion of the CRISPR array by PCR of both CR1 and CR3 loci, using the appropriate primers (Table S1). The plasmid source of the newly acquired spacers was confirmed by sequencing of the PCR products.

*Determining Relatedness of Phages*

All proteins were extracted from the publicly available complete phage genomes using in-house Python (https://www.python.org) and BioPython scripts (24). The proteins were then grouped in orthologous clusters using CogSoft (25) with an e-value threshold of 10^-5^. The output of the clustering analysis was then parsed with in-house python scripts to obtain a matrix where each column corresponds to a protein cluster and each row corresponds to a phage genome. If a genome has a gene that belongs to the cluster x, a value of 1 is given to the corresponding column otherwise a value of 0 is given. A similarity matrix was then generated by the *dist* function in R, using the binary method to calculate the distance (26). Finally, a heatmap for the relatedness was generated using the gplots package (27) in R.

*Construction of programming vectors*

Oligos were designed to match both strands of a protospacer shared by seven of the 13 *S. thermophilus* phages for which the genome is publicly available (Fig. S1), including an appropriate PAM and sites to facilitate cloning (Table S1). 25 nmol of each complementary oligo were held at 98 °C for 3 minutes, then cooled at a rate of 0.1 °C/s to a temperature of 50 °C to allow annealing of the oligos. The resulting dsDNA oligos were diluted to obtain a 1:3 vector:insert ratio with the XhoI/EcoRI-digested pNZ123. The vector and insert were incubated at 16 °C for 2 h in the presence of T4 DNA Ligase (Invitrogen, Ontario, Canada), and subsequently transformed into chemically competent NEB5α (New England Biolabs, Ontario, Canada) according to the manufacturer’s recommendations, and plated on LB with 20 μg/ml chloramphenicol (Cm 20).

Individual colonies were screened for presence of the desired insert by colony PCR with the pNZinsF/pNZinsR primers (Table S1), and colonies with correctly sized inserts were grown overnight at 37 °C in LB Cm 20 broth. Plasmids were purified from 3 ml of these cultures using a Qiagen Miniprep kit (Qiagen, Ontario, Canada) and their identity confirmed by sequencing with both pNZinsF and pNZinsR primers. The sequence-confirmed plasmids were then transformed into freshly prepared electrocompetent *S. thermophilus* DGCC7710. These cells were prepared by adapting a transformation protocol for *Lactococcus lactis* (28) in the following ways: replacing sucrose with 0.4 M sorbitol, GM17 media with LM17, and growing the cells at 42 °C rather than at 30 °C. The electroporated culture was plated on LM17 Cm 10 and incubated overnight at 37 °C. *S. thermophilus* DGCC7710 transformants were purified, and the presence of the plasmid confirmed by colony PCR using the above primers.

*Preparation and titration of phage lysates*

The host strain *S. thermophilus* DGCC7710 and phage 2972 were co-inoculated from freezer stocks in LM17 broth with 10 mM CaCl_2_, and incubated overnight at 42 °C. The resulting lysate was filtered (0.45 μm) and used to inoculate at 1% a fresh culture (OD_600_ of 0.1) of *S. thermophilus* DGCC7710 in LM17 broth with 10 mM CaCl_2_ and incubated overnight at 42 °C. This enriched lysate was then filtered with a 0.45 μm filter. This enriched lysate was titred using the double layer technique as previously described (15). Phage lysates were then freshly diluted in phage buffer (50mM Tris-HCl, pH 7.5, 100mM NaCl, 8mM MgSO4) for each assay so that a 1:3 phage:culture mix would result in a multiplicity of infection (MOI) of 5.

*Programming Assays*

*S. thermophilus* DGCC7710 with either the programming vector (pNZCR1, pNZCR3) or empty vector (pNZ123) were grown overnight in LM17 Cm 10 broth at 37 °C, and 100 μl of the overnight culture was used to inoculate an LM17 (selection-free) broth culture. In order to determine the culture density, once the culture reached an OD_600_ of 0.6 (~7 generations), in biological triplicate, a sample was serially diluted in fresh media and, in technical triplicate, 300 μl of the diluted culture was added to 3 ml of molten LM17 soft agar (0.75%) supplemented with 10 mM CaCl_2_, poured and set over a plate of 1% agar LM17 10 mM CaCl_2_. When conducting the programming assays, diluted phage lysate (see “*Preparation and titration of phage lysates”,* above) was mixed 1:3 with cultures of OD_600_ = 0.6 supplemented with 10 mM CaCl_2_, incubated for 5 min at 42 °C, and then serially diluted in fresh media. In triplicate, 400 μl the diluted phage:culture mix was then added to 3 ml of molten LM17 soft agar (0.75%) supplemented with 10 mM CaCl_2,_ poured and set over a plate of 1% agar LM17 10 mM CaCl_2._ The agar overlays were incubated overnight at 42 °C, after which surviving colonies were counted.

Surviving colonies were then screened for presence of the desired spacer in the CRISPR array of choice by colony PCR using a spacer-specific primer (Table S1; Rorf37_CR3, Rorf37_CR1). Colonies which had acquired the desired spacer were re-plated on both LM17 and LM17 Cm 10 to confirm loss of the plasmid-borne antibiotic resistance marker, and screened by colony PCR with primers pNZinsF/R to confirm loss of the plasmid. For colonies in which a desired spacer was not obtained, both the CR1 and CR3 arrays were amplified in a new spacer-independent fashion (Table S1; CR3-Rev, RDS7rev) to confirm expansion of at least one of the CRISPR arrays. Any PCR product with a size increase indicative of CRISPR array expansion was sent for sequencing to identify the newly acquired spacer(s).

**References :**

24 **Cock PJA, Antao T, Chang JT, Chapman BA, Cox CJ, Dalke A, Friedberg I, Hamelryck T, Kauff F, Wilczynski B, de Hoon MJL**. 2009. Biopython: freely available Python tools for computational molecular biology and bioinformatics. Bioinformatics **25**:1422–1423.

25 **Kristensen DM, Kannan L, Coleman MK, Wolf YI, Sorokin A, Koonin EV, Mushegian A**. 2010. A low-polynomial algorithm for assembling clusters of orthologous groups from intergenomic symmetric best matches. Bioinformatics **26**:1481–1487.

26 R: A language and environment for statistical computing (R Foundation for Statistical Computing, Vienna, Austria, 2011).

27 gplots: Various R programming tools for plotting data, R package version 2.14.0.

28 **Holo H, Nes IF.** 1989. High-frequency transformation, by electroporation, of *Lactococcus lactis* subsp. *cremoris* grown with glycine in osmotically stabilized media. Appl Environ Microb **55**:3119-3123.

**Figure S1:** Shared protospacer detection for all 13 complete *S. thermophilus* phage genomes currently available in public databases. The matrix (above) highlights the similarity of any phage to all others, as calculated by attributing increasing relatedness (darker colour) based on the number of protein ‘homologues’ they share (see Methods). By this method, *S. thermophilus* phages separate into at least three clearly distinct groups, identified by blue, white (none) or red shading. The table (below) displays all protospacers shared by the greatest number of phage genomes (≥7), and their presence (blue) or absence (white) from each phage genome. The bolded row outline indicates the two protospacers chosen for this study. S = Shortened spacer; querying our database with shorter and shorter spacer lengths, to a minimum of 15, only yielded one candidate with homology to more than 7 database phages.

**Table S1:** Strains, plasmids and oligos used in this study

| *Strains* | Species | Description | Source |
| --- | --- | --- | --- |
| NEB5α | *Escherichia coli* | Competent cells, cloning | NEB |
| DGCC7710 | *Streptococcus thermophilus* | Active & adaptive CRISPRs | (1) |
| SMQ-1333 | *E. coli* | NEB5α (pNZCR1), Cm^R^ | This study |
| SMQ-1334 | *S. thermophilus* | DGCC7710 (pNZCR1), Cm^R^ | This study |
| SMQ-1335 | *S. thermophilus* | DGCC7710 w/target CR1 spacer | This study |
| SMQ-1336 | *E. coli* | NEB5α (pNZCR3), Cm^R^ | This study |
| SMQ-1337 | *S. thermophilus* | DGCC7710 (pNZCR3), Cm^R^ | This study |
| SMQ-1338 | *S. thermophilus* | DGCC7710 w/target CR3 spacer | This study |
| SMQ-1339 | *S. thermophilus* | DGCC7710 (pNZ123), Cm^R^ | This study |
| *Plasmids* | Description | Function | Source |
| pNZ123 | Native vector, encodes chloramphenicol resistance | Negative control | (18) |
| pNZCR1 | pNZ123 with both CR1 oligos ligated in XhoI/EcoRI cut sites | CR1 programming | This study |
| pNZCR3 | pNZ123 with both CR3 oligos ligated in XhoI/EcoRI cut sites | CR3 programming | This study |
| *Oligos* | Sequence 5’-3’ | Function | Source |
| pNZins_F | AATGTCACTAACCTGCCC | pNZ123 insert screening | This study |
| pNZins_R | CATTGAACATGCTGAAGA | pNZ123 insert screening | This study |
| Forf37_CR1 | **TCGA**AGAAGCACCTCTTGCGTTGATAAAAGTATTGCAGAAA | pNZCR1 generation | This study |
| Rorf37_CR1 | **AATT**TTTCTGCAATACTTTTATCAACGCAAGAGGTGCTTCT | pNZCR1 generation, screening | This study |
| Forf37_CR3 | **TCGA**CCAATGACTGAAAACGACATTCGGAGGGTGTGGCG | pNZCR3 generation | This study |
| Rorf37_CR3 | **AATT**CGCCACACCCTCCGAATGTCGTTTTCAGTCATTGG | pNZCR3 generation, screening | This study |
| CR3-fwd | CTGAGATTAATAGTGCGATTACG | CR3 locus screening | (20) |
| CR3-rev | GCTGGATATTCGTATAACATGTC | CR3 locus screening | (20) |
| Yc70 | TGCTGAGACAACCTAGTCTCTC | CR1 locus screening | (20) |
| RDS7rev | GGATCCGGATCCGTTGAGGCCTTGTTC | CR1 locus screening | (5) |

**Bolded** text indicates overlaps to facilitate ligation into chosen site. Underlined text highlights PAM.

Cm^R^ is chloramphenicol resistance.

**References (Supplemental):**

24 **Cock PJA, Antao T, Chang JT, Chapman BA, Cox CJ, Dalke A, Friedberg I, Hamelryck T, Kauff F, Wilczynski B, de Hoon MJL**. 2009. Biopython: freely available Python tools for computational molecular biology and bioinformatics. Bioinformatics **25**:1422–1423.

25 **Kristensen DM, Kannan L, Coleman MK, Wolf YI, Sorokin A, Koonin EV, Mushegian A**. 2010. A low-polynomial algorithm for assembling clusters of orthologous groups from intergenomic symmetric best matches. Bioinformatics **26**:1481–1487.

26 R: A language and environment for statistical computing (R Foundation for Statistical Computing, Vienna, Austria, 2011).

27 gplots: Various R programming tools for plotting data, R package version 2.14.0.

28 **Holo H, Nes IF.** 1989. High-frequency transformation, by electroporation, of *Lactococcus lactis* subsp. *cremoris* grown with glycine in osmotically stabilized media. Appl Environ Microb **55**:3119-3123.
